# 7^th^ Brazilian Guideline of Arterial Hypertension: Chapter 4 - Cardiovascular Risk Stratification

**DOI:** 10.5935/abc.20160154

**Published:** 2016-09

**Authors:** MVB Malachias, MFT Neves, D Mion Júnior, GV Silva, HF Lopes, W Oigman

## Introduction

The global CV risk should be assessed in each hypertensive individual, because it aids the professionals in therapeutic decision-making and allows prognostic analysis. The identification of hypertensive individuals prone to CV complications, especially myocardial infarction (MI) and stroke, is fundamental to a more aggressive therapy. Several algorithms and risk scores based on population studies were created in past decades,^1^ but, considering the lack of Brazilian data for a more accurate assessment of CV risk in the Brazilian population, the use of one single risk score should be avoided to support therapeutic decisions. Multifactorial models of risk stratification can be used for a more accurate individual risk classification.

Informing patients about their RF can improve the efficacy of pharmacological and non-pharmacological measures to reduce global risk. In addition, estimating indicators and using aging-related terms, such as "vascular age" or "cardiometabolic age", can aid in the strategy to change the RF.^2,3^ See below some electronic addresses to estimate the vascular or cardiometabolic age recommended by American, Canadian and British societies.^4-6^

1. www.framinghamheartstudy.org/risk-functions/cardiovascular-disease/10-year-risk.php
  → supported by the National Heart, Lung, and Blood Institute and Boston University
2. www.nhs.uk/Conditions/nhs-health-check/Pages/check-your-heart-age-tool.aspx
  → supported by the British Heart Foundation
3. cardiometabolicage.com
  → supported by the Canadian Institute for Health Research (CIHR) and McGill University

In clinical practice, CV risk stratification of hypertensive patients can be based on two different strategies. In the first, the assessment is aimed at determining the global risk directly related to hypertension, in which case the risk classification depends on BP levels, associated risk factors, TOD and presence of CVD or kidney disease. In the second strategy, the objective is to determine the risk of a certain individual to develop general CVD within 10 years. Although that form of assessment is not specific to hypertensive patients, since it can be applied to any individual aged 30-74 years, it is worth noting that AH is the major CVRF.

### Additional cardiovascular risk stratification

Only a small minority of hypertensive patients has only one BP elevation. Aimed at making risk stratification easier, the classification system in Table 1, contemplating only low, moderate and high risk, should be used. It is worth noting that the identification of previous CVD, kidney disease or DM considerably increases the risk of future CV events, independently of BP levels.^7,8^

**Table 1:** Risk stratification in hypertensive patients based on additional risk factors, presence of target-organ damage and cardiovascular or kidney disease

	SBP 130-139 orDBP 85-89	Stage 1 SAHSBP 140-159 or DBP 90-99	Stage 2 SAHSBP 160-179 or DBP 100-109	Stage 3 SAHSBP ≥ 180 or DBP ≥ 110
No risk factor	No additional risk	Low Risk	Intermediate risk	High Risk
1-2 risk factors	Low Risk	Intermediate risk	High Risk	High Risk
≥ 3 risk factors	Intermediate risk	High Risk	High Risk	High Risk
Presence of TOD, CVD, CKD or DM	High Risk	High Risk	High Risk	High Risk

SBP: systolic blood pressure; DBP: diastolic blood pressure; SAH: systemic arterial hypertension; CVD: cardiovascular disease; CKD: chronic kidney disease; DM: diabetes mellitus: TOD: target-organ damage.

The large majority of the hypertensive population has additional RF. Therefore, the CV risk assessment depends on information obtained from clinical history, physical examination and complementary tests, always aiming at:

- Coexistence of other CVRF (Table 2);
  **Table 2:** Cardiovascular risk factors in the assessment of additional risk in hypertensives

• Male sex
• Age
○ Men ≥ 55 years or women ≥ 65 years
• History of premature CVD in first-degree relatives
○ Men < 55 years or women < 65 years
• Smoking habit
• Dyslipidemia
○ Total cholesterol > 190 mg/dL and/or
○ LDL-cholesterol > 115 mg/dL and/or
○ HDL-cholesterol < 40 mg/dL in men or < 46 mg/dL in women and/or
○ Triglycerides > 150 mg/dL
• Insulin resistance
○ Fasting serum glycemia: 100-125 mg/dL
○ Oral glucose tolerance test: 140-199 mg/dL in 2 hours
○ Glycated hemoglobin: 5.7 – 6.4%
• Obesity
○ BMI ≥ 30 kg/m^2^
○ AC ≥ 102 cm in men or ≥ 88 cm in women

CVD: cardiovascular disease; LDL: low-density lipoprotein; HDL: highdensity lipoprotein; BMI: body mass index; AC: abdominal circumference.
- Presence of hypertension TOD (Table 3);
  **Table 3:** Target-organ damage in the additional risk assessment of hypertensives

• Left ventricular hypertrophy
○ ECGI: Sokolow-Lyon index (SV_1_ + RV_5 _or RV_6_) ≥ 35 mm
○ ECGI: R aVL > 11 mm
○ ECGI: Cornell voltage > 2440 mm*ms
○ ECHOI: LVMI > 115 g/m^2^ in men or > 95 g/m^2^ in women
• Carotid IMT > 0.9 mm or carotid plaque
• Carotid-femoral PWV > 10 m/s
• ABI < 0.9
• Stage 3 chronic kidney disease (GFR 30-60 mL/min/1.73m^2^)
• Albuminuria = 30 - 300 mg/24h or UACR = 30 - 300 mg/g

ECG: electrocardiogram; ECHO: echocardiogram; IMT: intima-media thickness; LVMI: left ventricular mass index; PWV: pulse wave velocity; ABI: ankle-brachial index; GFR: estimated glomerular filtration rate; UACR: urine albumincreatinine ratio.
- Diagnosis of CVD or kidney disease already established (Table 4).
  **Table 4:** Established cardiovascular and kidney disease in the additional risk assessment of hypertensives.

• Cerebrovascular disease
○ Ischemic stroke
○ Cerebral hemorrhage
○ Transient ischemic attack
• Coronary artery disease
○ Stable or unstable angina
○ Myocardial infarction
○ Myocardial revascularization: percutaneous (angioplasty) or surgical
○ Heart failure with reduced or preserved ejection fraction
○ Symptomatic peripheral arterial disease of lower limbs
○ Stage 4 chronic kidney disease (GFR < 30 mL/min/1.73m^2^) or albuminuria > 300 mg/24h
○ Advanced retinopathy: hemorrhages, exudates, papilledema

GFR: estimated glomerular filtration rate.

**Figure 1:**
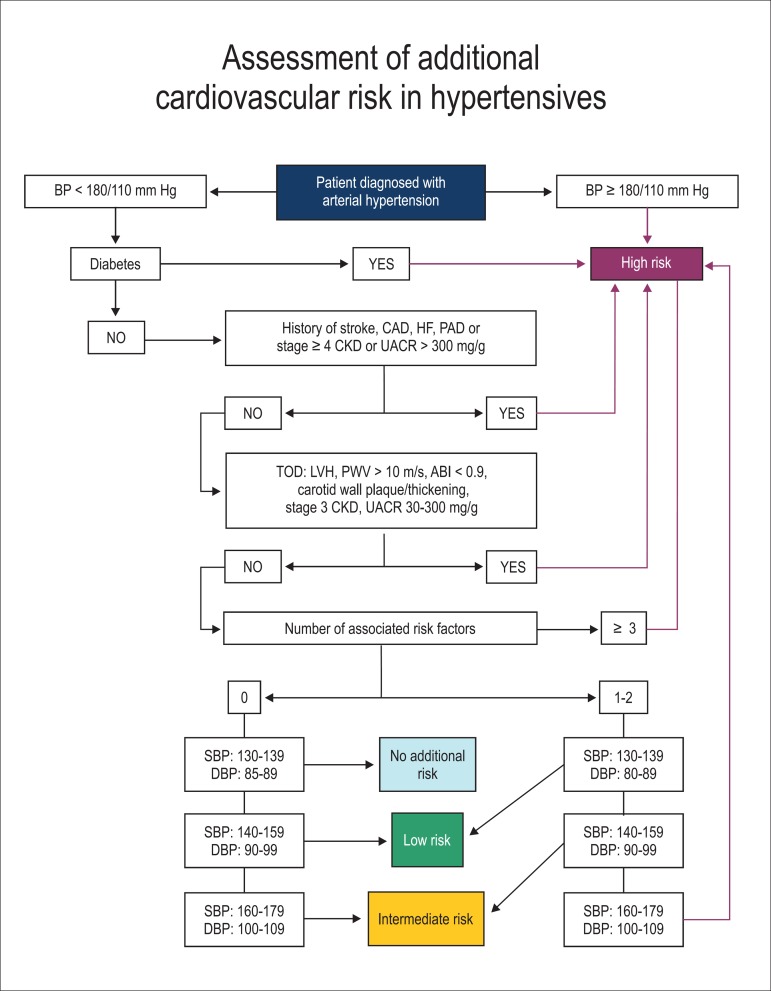
Flowchart of classification of additional CV risk for hypertensive patients. BP: blood pressure; CAD: coronary artery disease; HF: heart failure; PAD: peripheral arterial disease; CKD: chronic kidney disease; UACR: urine albumin/creatinine ratio; TOD: target-organ damage; LVH: left ventricular hypertrophy; PWV: pulse wave velocity; ABI: ankle-brachial index; SBP: systolic blood pressure; DBP: diastolic blood pressure. Risk factors: male sex, age > 55 years (men) or > 65 years (women), family history, smoking, dyslipidemia, obesity and insulin resistance.

Thus, to facilitate and speed the classification process of additional CV risk in the medical visit setting, the health professional in charge should follow the flowchart described in Figure 1. It is worth noting that, in some cases, the initial classification can be modified according to the best or worst control of BP levels and RF.

### Global cardiovascular risk stratification

The CV risk stratification based on three steps has been recently recommended in the V Brazilian Guideline for Dyslipidemia and Atherosclerosis Prevention^9^ and the I Brazilian Guideline for Cardiovascular Prevention,^10^ and it can be adopted for hypertensive patients. The steps should be performed as follows.

### Identification of atherosclerotic disease or of its equivalents

The first step to estimate CV risk is the identification of clinically evident or subclinical atherosclerotic disease, or of its equivalents, such as DM and CKD^11^ (Table 5). If positive, the individual is immediately classified as at high risk, because the chance of having the first or a new CV event within 10 years is greater than 20%. (GR: I; LE: A).

**Table 5:** Definition of atherosclerotic disease and of its equivalents

1. Atherosclerotic disease (clinically evident): coronary, cerebrovascular or peripheral obstructive disease
2. Significant subclinical atherosclerosis documented by use of diagnostic methods
3. Arterial revascularization procedures
4. Types 1 and 2 diabetes mellitus
5. Chronic kidney disease
6. Family hypercholesterolemia

### Global risk score analysis

When the individual does not meet any of the step 1 conditions, the next step is to estimate the Global Risk Score (GRS).^6^ The algorithm estimates the risk of having a CV event (CAD, stroke, PAD, HF) within 10 years. The distribution of points and percentage of risk is differentiated for women (Tables 6A and 6B) and men (Tables 7A and 7B). When the GRS is lower than 5%, the patient is classified as 'low risk' (GR: A; LE: I), except those with a family history of premature CV disease, who are reclassified as 'intermediate risk'. (GR: IIa; LE: B).

**Table 6(A):** Points in the global risk score for women

Points	Age (years)	HDL-C	TC	SBP (non-treated)	SBP (treated)	Smoking	Diabetes
-3				< 120			
-2		60+					
-1		50-59			< 120		
0	30-34	45-49	< 160	120-129		No	No
1		35-44	160-199	130-139			
2	35-39	< 35		140-149	120-129		
3			200-239		130-139	Yes	
4	40-44		240-279	150-159			Yes
5	45-49		280+	160+	140-149		
6					150-159		
7	50-54				160+		
8	55-59						
9	60-64						
10	65-69						
11	70-74						
12	75+						

HDL-C: high-density lipoprotein cholesterol; TC: total cholesterol; SBP: systolic blood pressure.

**Table 6(B):** Global CV risk for women according to the points obtained

Points	Risk (%)	Points	Risk (%)
≤ -2	< 1	10	6.3
-1	1.0	11	7.3
0	1.2	12	8.6
1	1.5	13	10.0
2	1.7	14	11.7
3	2.0	15	13.7
4	2.4	16	15.9
5	2.8	17	18.5
6	3.3	18	21.6
7	3.9	19	24.8
8	4.5	20	28.5
9	5.3	21+	>30

**Table 7(A):** Points in the global risk score for men

Points	Age (years)	HDL-C	TC	SBP (non-treated)	SBP (treated)	Smoking	Diabetes
-2		60+		< 120			
-1		50-59					
0	30-34	45-49	< 160	120-129	< 120	Não	Não
1		35-44	160-199	130-139			
2	35-39	< 35	200-239	140-159	120-129		
3			240-279	160+	130-139		Sim
4			280+		140-159	Sim	
5	40-44				160+		
6	45-49						
7							
8	50-54						
9							
10	55-59						
11	60-64						
12	65-69						
13							
14	70-74						
15+	75+						

HDL-C: high-density lipoprotein cholesterol; TC: total cholesterol; SBP: systolic blood pressure.

**Table 7(B):** Global CV risk for men according to the points obtained

Points	Risk (%)	Points	Risk (%)
≤ -3	< 1	8	6.7
-2	1.1	9	7.9
-1	1.4	10	9.4
0	1.6	11	11.2
1	1.9	12	13.2
2	2.3	13	15.6
3	2.8	14	18.4
4	3.3	15	21.6
5	3.9	16	25.3
6	4.7	17	29.4
7	5.6	18+	> 30

Men with GRS between 5% and 20%, and women with GRS between 5% and 10% are initially considered at 'intermediate risk'.^12^ (GR: I; LE: A).

Men with GRS > 20% and women with GRS > 10% are considered at 'high risk' (GR: I; LE: A).

### Risk reclassification based on the presence of aggravating factors

Patients at intermediate risk with the aggravating factors listed in Table 8 are reclassified as at high risk.^9,13-15^ (GR: IIa; LE: B).

**Table 8:** Aggravating factors of CV risk

Aggravating factor	Recommendations and evidence
1. Family history of premature CAD in first-degree relative, men < 55 years or women < 65 years	GR: IIa; LE: A
2. Diagnosis of MS according to the IDF criteria	GR: IIb; LE: A
3. Microalbuminuria (30-300 mg/g creatinine) or albuminuria (> 300 mg/g creatinine)	GR: IIa; LE: B
4. LVH	GR: IIa; LE: B
5. High-sensitive C-reactive protein > 2 mg/L	GR: IIa; LE: B
6. Carotid IMT > 1.0 mm	GR: IIb; LE: B
7. Coronary calcium score > 100 or > 75^th^ percentile for age and sex	GR: IIa; LE: A
8. ABI < 0.9	GR: IIa; LE: A

CAD: coronary artery disease; MS: metabolic syndrome; IDF: International Diabetes Federation; LVH: left ventricular hypertrophy; IMT: intima-media thickness; ABI: ankle-brachial index.

The criteria used in the diagnosis of MS are shown in Table 9.

**Table 9:** Diagnostic criteria for metabolic (syndrome defined with 3 or more criteria)15,16

Criteria	Definition
1. Abdominal obesity	
Men	≥ 94 cm
Women	≥ 80 cm
2. HDL-cholesterol	
Men	< 40 mg/dl
Women	< 50 mg/dl
3. Triglycerides (or treatment for hypertriglyceridemia)	≥ 150 mg/dl
4. BP (or treatment for arterial hypertension)	
SBP and/or	≥ 130 mmHg
DBP	≥ 85 mmHg
5. Glycemia (or treatment for DM)	≥ 100 mg/dl

BP: blood pressure; SBP: systolic blood pressure; DBP: diastolic blood pressure; DM: diabetes mellitus.

**Figure 2:**
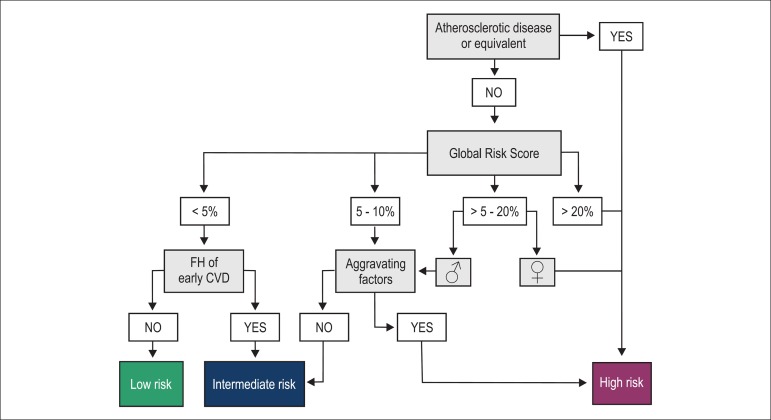
Flowchart to estimate global cardiovascular risk. FH: family history; CVD: cardiovascular disease.

In addition, to facilitate the global CV risk determination in hypertensive patients, the flowchart in Figure 2 shows all steps necessary for the final classification.

In conclusion, so far no CV risk assessment way has been validated in Brazil. In addition, some young women tend to a risk estimate lower than the actual one, and older men are usually identified as at high risk, even with no relevant RF. Thus, the use of more than one classification allows better understanding of CV risk in hypertensive patients.
